# Metabolomic profile of amniotic fluid to evaluate lung maturity: the diaphragmatic hernia lamb model

**DOI:** 10.1186/2049-6958-9-54

**Published:** 2014-11-04

**Authors:** Gloria Pelizzo, Maurizio Ballico, Maria Chiara Mimmi, José Louis Peirò, Mario Marotta, Costanzo Federico, Erika Andreatta, Ghassan Nakib, Maurilio Sampaolesi, Elisa Zambaiti, Valeria Calcaterra

**Affiliations:** Department of the Mother and Child Health, Pediatric Surgery Unit, Fondazione IRCCS Policlinico San Matteo, Pavia and University of Pavia, Piazzale Golgi 2, 27100 Pavia, Italy; Department of Medical and Biological Sciences, University of Udine, Udine, Italy; Cincinnati Fetal Center. Pediatric Surgery Division, CCHMC, Cincinnati, OH USA; Fetal Surgery Program, Congenital Malformations Research Group, Research Institute of Hospital Universitari Vall d’Hebron, Edifici Infantil, Barcelona, Spain; Laboratory of Translational Cardiomyology, Stem Cell Interdepartmental Institute, KU Leuven and Human Anatomy, University of Pavia, Pavia, Italy; Department of Internal Medicine, University of Pavia, Pavia, Italy

**Keywords:** Amniotic fluid, Animal model, Congenital diaphragmatic hernia, Metabolomic, Tracheal occlusion

## Abstract

**Background:**

Tracheal occlusion (TO) stimulates lung growth in fetuses affected with congenital diaphragmatic hernia (CDH) although the processes involved in lung maturation still remain unknown. The objective of this study was to evaluate the metabolomic profile of amniotic fluid (AF) following TO in fetal lamb model in order to obtain an indirect view of mechanisms involved in pulmonary reversal hypoplasia and biochemical maturity in response to fetal TO.

**Methods:**

Liquid Chromatography Mass Spectrometry was performed on lamb AF samples at: age I (70 days’ gestation); age II (102 days’ gestation); age III (136 days’ gestation). CDH was induced at age I and TO at age II.

**Results:**

Betaine, choline, creatinine were found significantly increased during gestation in the control group. The CDH group showed choline (p =0.007) and creatinine (p =0.004) decreases during pregnancy. In the TO group choline and creatinine profiles were restored.

**Conclusions:**

Alveolar tissue and fetal global growth ameliorated after TO. Metabolomics provided useful information on biochemical details during lung maturation. Metabolomic profiling would help to identify the best time to perform TO, in order to increase survival of CDH affected patients.

## Background

Congenital diaphragmatic hernia (CDH) is a congenital birth defect that occurs in 1/2000 to 1/3000 newborns with a survival rate of 42-68%. Abnormal development of the pulmonary parenchyma and vasculature lead to variable degrees of respiratory insufficiency and pulmonary hypertension in early neonatal life [1–4].

While modern neonatal intensive care has improved the prognosis for surviving cases, comorbidities affect almost half of live-born infants and the mortality rate remains high in patients with poor prenatal prognosis. Experimental studies could potentially ameliorate outcomes in pulmonary hypoplasia [5, 6]. Fetal endoscopic tracheal occlusion (FETO) may still be a possible therapeutic procedure in case of severe CDH, even if the mortality rate is reported to be nearly 40% [7–9].

Amniotic fluid (AF) composition reflects the physiological status during fetal development and it may be used to detect potential pathological conditions. AF contains large amounts of proteins and metabolites produced by the amnion epithelial cells, fetal tissues, fetal excretions and placental tissues.

Metabolomics is commonly used to measure the entire metabolomic profile in biological fluids, cells, tissues or organs and to elucidate the association between metabolic pathways and perturbations that arise as a result of disease or organ malformation [10–13].

The objective of this study was to determine amniotic fluid metabolic profiling changes in fetal lambs who underwent surgically induced CDH and subsequent tracheal occlusion (TO) which provides an excellent vision of lung biochemical maturity and is an excellent model to determine the real benefits and best timing for TO.

## Methods

The experimental protocol was approved by the National Animal Care and Ethics Committee and was conducted in accordance with Italian and European legislation (D.lgs. 116/92, European Directives 86/609/EE for the protection of animals used in scientific and experimental studies and 2010-63UE).

A preliminary study with Liquid Chromatography Mass Spectrometry (LC-MS) [14, 15] was performed on 20 lamb AF samples, taken at three gestational ages: Age I (70 days’ of gestation); Age II (102 days’ gestation); Age III (136 days’ gestation).

CDH induction was performed at Age I and TO was carried out during the 2^nd^ trimester at Age II.

Based on this information, AF samples were divided into:

– **CONTROL group**, healthy control pregnancies: Age I, number of samples =5; Age II, number of samples =3; Age III, number of samples =3;
– **CDH group**, fetuses with induced CDH: Age II, number of samples =3 and at Age III, number of samples =3;
– **CDH + TO group**, fetuses with induced CDH and TO treatment: Age III, number of samples =3

Healthy control AF samples were collected to study the normal metabolomic evolution.

We report on the effect of induced CDH and TO treatment for selected metabolites at stage Age III.

### Anesthesia protocol

All surgical procedures were performed under general anesthesia. After premedication with intramuscular (IM) injection of 5 mg/Kg ketamine and 0.2 mg/Kg midazolam (Dormicum®, Roche), intravenous access (IV) was established at the external jugular vein.

The pregnant ewes were pre-oxygenated using a facemask and induced with 5 mg/Kg IV propophol (Propofol®-Lipuro 1%, B. Braun Melsungen AG). Endotracheal intubation was performed, and general anesthesia was maintained with isoflurane 2% (Isoflo, Abbott laboratories Ltd) in 100% oxygen (1.5 L/minute).

Esophageal intubation was performed to prevent ruminal bloat. A continuous infusion of Ringer lactate (B. Braun Melsungen AG) was administered at 10 ml/Kg/h during surgery. Intra-operative monitoring consisted of electrocardiography, pulse oximetry, non invasive blood pressure and capnography. All animals received a 75 μg transdermal fentanyl patch (Durogesic®, Janssen laboratories) for post-operative pain relief. For peri-operative infection prophylaxis, the animals received a single dose of 22 mg/Kg IV cephazolin (Kurgan®, Normon Laboratories) at the time of induction and 15 mg/Kg IM amoxicillin (Duphamox L.A., Fort Dodge) every 48 h, for eight days. Fetuses were anesthetized through the placenta and additional anesthesia with fentanil (10 μg/kg IM) and the muscle relaxant pancuronium (0.3 mg/Kg IM) were administered. Meloxicam 0.5 mg/kg IM /24 h was administered as post-operative analgesia for seven days. Maternal body temperature was monitored by using a digital probe and maintained at 36-37°C with a warming bed. Standard ECG electrodes were used for monitoring.

### Surgical technique

As a first step, malformation was surgically induced (day 70 of gestation). A midline laparotomy exposed the gravid uterus. The fetus was partially extruded from the uterus with a short hysterectomy (4 cm). A left fetal thoracotomy was achieved and a diaphragmatic hernia was created. The fetal stomach was raised into the thorax, which was closed into one layer. The fetus was returned to the uterus (2 gr of amoxicillin were added to the amniotic fluid). The uterus wall and maternal laparotomy were closed.

The next step included the tracheal occlusion (day 102 of gestation). The uterus was externalized after a midline laparotomy. The fetal lamb’s mouth was located and also partially externalized by a short hysterectomy (2 cm). A latex detachable balloon was placed into the trachea and inflated. The fetus was returned to the uterus and 2 gr of amoxicillin were added to the amniotic fluid. Closure of the uterus and the abdomen walls was performed.

At the end of the pregnancy (day 136 of gestation), lambs were delivered via terminal caesarean section, approximately 10 days before term to prevent natural parturition. Lambs were euthanized with a bolus of Pentobarbital 200 mg/Kg IV.

### Metabolomic analysis

#### Sample preparation

Amniotic fluid samples were centrifuged after collection in order to remove cells and cellular debris. The supernatants were immediately frozen and stored at -70°C/-80°C until metabolite extraction and analysis were performed. Supernatant samples were thawed at room temperature and methanol extraction was accomplished with the protocol reported by Graca et al. [16]. An internal standard (2’-deoxyadenosine) was added to the extracted dry aliquots which were reconstituted in ultrapure H_2_O (Milli-Q H_2_O).

#### HPLC-TOF-MS analysis

LC-MS analysis was performed by positive ionization mass spectrometry using a Q-STAR mass spectrometer (AB-SCIEX, Foster City, CA, USA) equipped with an electrospray ionization source; acquired data were analyzed using AB-SCIEX Analyst™ QS software (version 1.1). The HPLC system includes an Agilent 1100 series micro LC pump. The liquid chromatographic separation was performed on a Jupiter® 5 μm 300 Å (150 × 0.5 mm) Reverse PhaseC18 column (PhenomenexInc, Torrance, CA, USA) at a flow of 10 μL/min using a gradient from 0% to 90% of solvent Bover 35 min. Solvents A and Bconsisted of water and acetonitrile, respectively; both contained 0.1% (v/v) formic acid. All samples were analyzed in triplicate.

Metabolites were quantified using LC-MS-TOF standard runs in positive-ion mode, while LC-MS/MS runs were used for molecule identification. The assignment of the metabolites was based on analytical standards analyzed under the same chromatographic conditions.

### Data processing and analysis

The metabolite peaks from raw HPLC-MS chromatograms were integrated with the software Analyst QS 1.1 (Applied Biosystems-SCIEX) by evaluating the extracted ion chromatogram (XIC) counts. All data sets were normalized to account for variable sample dilution and experimental influences using the peak area of the internal standard (2’-deoxyadenosine).

The HPLC-MS data shown in Tables 1, 2 and 3 represent the metabolite XIC areas, which do not account for concentrations, but can be used to evaluate the relative level of each metabolite among the three sample groups (Control Age I/II/III, CDH Age II/III and CDH + TO Age III).

**Table 1:** **Metabolite MS peak area (Intensity Counts) in ovine amniotic fluid from a fetal lamb model of congenital diaphragmatic hernia (CDH) and tracheal occlusion (TO)**

MS peak area						
	Age I (70 days)	Age II (102 days)		Age III (136 days)		
Metabolite	Control	Control	CDH	Control	CDH	CDH + TO
Betaine	7931	19140	17905	91450	17210	27990
	±	±	±	±	±	±
	426	750	944	2646	566	901
Choline	1500	4793	3667	6281	1639	3933
	±	±	±	±	±	±
	179	381	118	375	146	14
Creatinine	687	19988	17115	189533	20330	118833
	±	±	±	±	±	±
	77	968	609	7821	495	3612

Data are expressed as group means ± standard deviation.

**Table 2:** **Metabolite MS peak area (Intensity Counts) in Age II ovine amniotic fluid**

Age II AF	MS peak area		Fold Difference	p
Metabolite	Control	CDH		
Betaine	19140	17905	1.07	0.089
	±	±		
	750	944		
Choline	4793	3667	1.31	0.007
	±	±		
	381	118		
Creatinine	19988	17115	1.17	0.004
	±	±		
	968	609		

Data are expressed as group means ± standard deviations. p refers to Welch’s ANOVA.

**Table 3:** **Metabolite MS peak area (Intensity Counts) in Age III ovine amniotic fluid**

Age III AF	MS peak area		Fold difference	Adjusted p	t	MS peak area		Fold difference	Adjusted p	t	MS peak area		Fold difference	Adjusted p	t
Metabolites	Control	CDH				Control	CDH + TO				CDH + TO	CDH			
Betaine	91450	17210	5.31	0.000	47.02	91450	27990	3.27	0.000	39.33	27990	17210	1.63	0.001	16.43
	±	±				±	±				±	±			
	2646	566				2646	901				901	566			
Choline	6281	1639	3.83	0.001	19.36	6281	3933	1.60	0.015	10.84	3933	1639	2.40	0.042	22.10
	±	±				±	±				±	±			
	375	146				375	14				14	146			
Creatinine	189533	20330	9.32	0.001	37.36	189533	118833	1.60	0.002	14.21	118833	20330	5.84	0.001	46.59
	±	±				±	±				±	±			
	7821	495				7821	3612				3612	495			

Data are expressed as group means ± standard deviation. Adjusted p and t were obtained with the One-way ANOVA with Games-Howell Pairwise Comparisons.

A comparison of Age II groups (Control II and CDH II) and among Age III groups (Control III, CDH III and CDH + TO III) was performed and the One-Way Analysis of Variance (ANOVA) was applied to determine whether the means of groups significantly differed. We performed Welch’s ANOVA, which does not assume constant variance across all groups, with an alpha level set at 0.05. In the case of Age III, sample differences between all pairs of groups were compared according to the Games-Howell method, which does not assume constant variance while controlling the multiple comparisons error rate.

## Results

The AF analysis in control samples (gestational Ages I to III) led to the identification of potentially interesting molecules in the amniotic fluid, including amino acids, organic acids, sugars and nucleotide metabolites. Among these, we observed that only three metabolites,betaine, choline and creatinine, continuously increased during ovine gestation, while all the other identified metabolites exhibited a variable trend including increasing and decreasing phases. This made the mentioned metabolites the best biomarkers of a physiological pregnancy and we therefore focused on these three compounds and analysed their variation following induced CDH and TO treatment.

**Figure 1:**
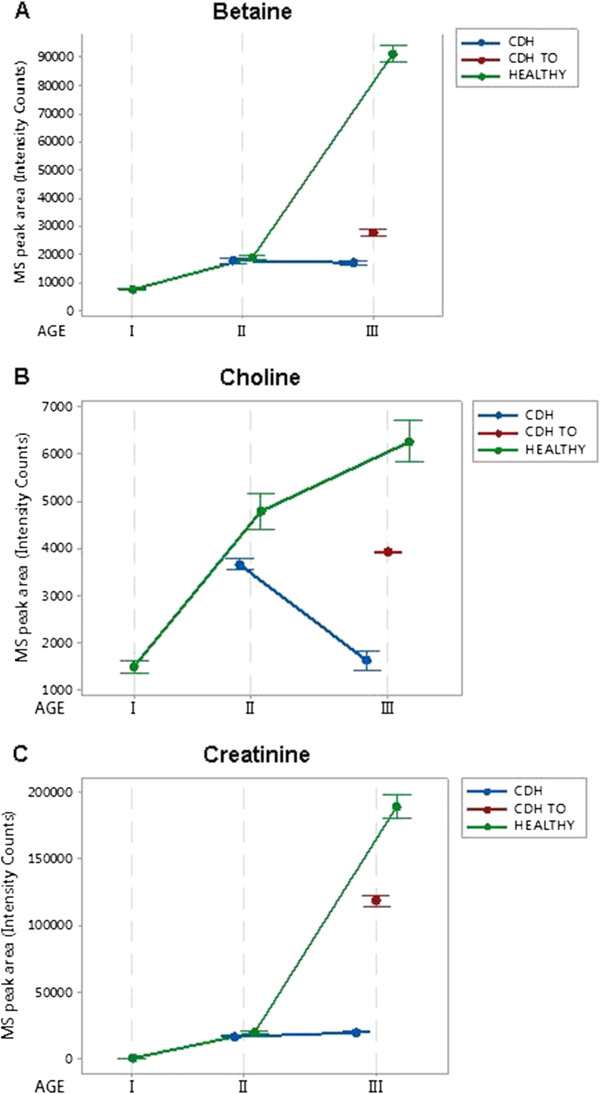
**Metabolomic data summarized in Table** 1 **.** The Interval Plots show the trend exhibited by the three metabolic markers: betaine (panel **A**), choline (panel **B**) and creatinine (panel **C**) during gestation in the case of “control/CDH induced/CDH induced and TO” treatedfetal lambs. Bullets represent the group mean values, while the interval bars extend 2 standard errors away from the mean.

According to the ANOVA analysis of age II groups (102 days), CDH induces a significant decrease in choline (p 0.007) and creatinine (p 0.004), while betaine alterations were not significant (p 0.089) (Figure 1 and Table 1).

The most significant alterations observed were in AF collected at Age III (136 days), since at this stage the effect of CDH could be evaluated either with or without the TO treatment.

The results from non-treated CDH, in comparison with healthy controls, resulted in the following adjusted p values: choline (p 0.001), creatinine ( p 0.001) and betaine (p 0.000) (Figure 1 and Table 2).

Interestingly, the TO treatment led to a substantial recovery in choline and creatinine levels and to a modest recovery in betaine (Figure 1). The ANOVA analysis of the Age III groups and the following Games Howell multiple comparison test are consistent with this trend (Figure 1 and Table 3). In particular, p obtained when comparing the control and CDH + TO groups indicate that by the end of gestation, betaine, choline and creatinine were positively affected by the TO treatment and resulted closer to the physiological level. In fact, although the p indicate a significant difference between the control and CDH + TO conditions, the comparison of fold difference values between control and CDH samples, with and without TO, show a significant recovery of the biomarkers associated with the TO treatment (Table 3).

## Discussion

Amniotic fluid volume and composition are the result of the dynamic interaction between the secretion of fetal lung liquid, fetal urine and the removal of fluid by fetal swallowing and/or resorption through the fetal membranes [17]. A great amount of AF comes from epithelial lung. Between 47-54% of the lung secreted fluids comes out of fetal trachea and are swallowed and the rest is mixed with the amniotic fluid during breathing movements [17].

Metabolomics of human AF has been recently used to detect different fetal pathologies in pregnancy such as chromosomal disorders [18–20], renal dysfunction [10, 21], metabolic disease [10], neural tube anomalies [10, 22–24] and defects of intrauterine growth [25–28]. Recent advances in metabolomics [10–13, 29] also introduce AF evaluation as a new approach to fetal pulmonary development studies.

Preliminary data of our study show an interesting increase in choline, betaine and creatinine levels during physiologic pregnancy. The profile of these metabolites increases constantly during gestational age. These molecules are detectable in AF in different concentrations, according to the gestational period [11, 12]. Changes in the gestational choline profile have already been correlated with the fetal lung maturity [30]. Choline seems to be involved in the synthesis of phospholipid, a major surfactant component. The role of betaine is unclear; it is formed by choline oxidation catalyzed by the mitochondrial enzyme choline dehydrogenase [16, 30, 31]. Betaine is also engaged in pulmonary development in the same metabolic pathway as choline. Creatinine has also been used long as a test of fetal maturity. Bock showed that in relation to maturity of the fetus, the relative intensity of the creatinine peak correlated best with conventional tests of fetal lung maturity [12]. Choline and creatinine are involved in development of different fetal organs. The possibility that the profile of these metabolites may be influenced by other variables, such as the fetal renal system, cell membrane turnover, cannot be excluded.

Newborns with CDH have hypoplastic lung. These patients have normal maturation values for alveolar epithelial type II. At birth, surfactant and phospholipid production is not delayed [31, 32]; the total amount of cells is reduced, as expected in a hypoplasia condition. Prenatal TO to treat severe CDH seems to improve pulmonary growth [1–4, 33, 34]. TO prevents egress of pulmonary fluid leading to lung tissue stretching and reversal of lung hypoplasia. The lung parenchymal structure increases in alveolar growth and inter-alveolar wall thickness. In the animal model, sustained FETO causes type II cell depletion resulting in significantly low surfactant production. Temporary FETO fails to cause lung growth in the CDH model, but it preserves type II cells, surfactant production, and partially corrects abnormal muscularization of pulmonary arterioles seen in pulmonary hypertension [31–36].

Our study confirms the perturbation of lung maturity in CDH. Certain AF metabolites stop increasing from stage II to stage III in CDH fetuses. The intensity peaks of creatinine and betaine at stage III, at the end of gestation, were similar to those found at stage II but reduced in average compared to controls at the same gestational age (stage II). Choline intensity peaks in CDH fetuses were even found to be significantly lower during gestation than control specimens; growth curve did not increase from the beginning of gestation and values found at term were lower than those found in controls at stage I.

In this study, sustained TO was performed to reproduce the same conditions induced by FETO in CDH human fetuses. Surprisingly, fetal TO seems to improve the profile of the three metabolites: immediately at Age II after TO induction and at stage III gestation. Creatinine had the most improved metabolomic recovery profile, supporting the role of this metabolite as a marker of lung involvement in fetal development [12, 30].

The mild response of betaine, with respect to creatinine and choline, could be related to the involvement of this metabolite in simultaneous methylation reactions which also include the development of fetal respiratory system.

Nuclear magnetic resonance (NMR) spectroscopy or mass spectrometry (MS) are employed to characterize metabolomic profiles and quantify all small molecules in biological samples. Thus a comprehensive global monitoring of metabolites and their fluctuations in response to various stimuli can be achieved. Metabolomic profiling can provide information on what is actually changing in a biological system, and serves as a crucial link between phenotype and genetics [13–16]. Simultaneous analysis of different groups of metabolic markers like amino acids (*ie* alanine), sugars (*ie* glucose) and muscle catabolites (*ie* creatinine) in a single MR spectrum allows indirect assessment of function and development of fetal organs [11–13]. The observed variation could also reflect the AFS cell composition in the AF. In this view, congenital diaphragmatic hernia could trigger AFS cell maturation according to tissue regeneration needs, resulting in a AF metabolic signature in the CHD lamb model [9].

There are no previous studies on the metabolomic profile of the amniotic fluid in the CDH lamb model after TO.

This approach provided an indirect index of fetal lung maturity, but the involvement of surfactant production of type II cells needs to be verified in further studies. Concomitant metabolomics, histological and immunochemical studies, are needed to confirm a direct correlation between AF profile, type II pneumocyte integrity and lung maturation before and after TO.

Although this is a preliminary study, our findings support the hypothesis that the best biomarkers of a physiological pregnancy may be considered as suggestive indexes of lung maturity also in CDH fetuses. Increased knowledge of pulmonary maturation may be useful in defining biochemical mechanisms which are at the basis of lung hypoplasia in CDH fetuses. The effects of TO confirm their role in restoring the processes involved in surfactant-mediated lung maturation. This study also supports novelinvestigations in potential pathways implicated in AF regulatory mechanisms. Intra-tracheal pulmonary absorption could be considered an influent pathway in the amniotic fluid dynamics.

## Conclusions

AF metabolomic profiles may be considered indirect markers of lung growth and could be useful in defining the prognosis of CDH fetuses. Metabolomic analysis in AF in a CDH animal model, provides useful information on fetal lung biochemical mechanisms involved in pulmonary development processes.

Further investigations are still needed to identify new biochemical macromolecules involved in fetal lung maturation to improve survival of severe CDH fetuses.

## Abbreviations

AF: Amniotic fluid
AFS: Amniotic fluid-derived stem
CDH: Congenital diaphragmatic hernia
FETO: Fetal endoscopic tracheal occlusion
NMR: Nuclear magnetic resonance
MS: mass spectrometry
TO: Tracheal occlusion
VEGF: Vascular endothelial growth factor.
